# A proteomics-based method for identifying antigens within immune complexes

**DOI:** 10.1371/journal.pone.0244157

**Published:** 2020-12-23

**Authors:** Stephanie Menikou, Andrew J. McArdle, Ming-Shi Li, Myrsini Kaforou, Paul R. Langford, Michael Levin

**Affiliations:** Department of Infectious Disease, Section of Paediatric Infectious Disease, Imperial College London, London, United Kingdom; New York State Department of Health, UNITED STATES

## Abstract

A novel approach to recover and identify immune complexes (ICs) was developed using size exclusion chromatography (SEC) and affinity chromatography on immunoglobulin binding columns (HiTrap Protein G). The purification process was monitored by 1D SDS-PAGE, protein staining, Western blotting and, finally, liquid chromatography tandem mass spectrometry (LC MS/MS) was used to identify the recovered antigens. This approach was applied to serum with artificially created immune complexes (ICs) comprising vaccine antigen (influenza) and antibody, which led to recovery and identification of influenza peptides within the recovered ICs. This approach was compared with the established method for IC detection and recovery, polyethylene glycol (PEG) precipitation, followed by LC MS/MS. Both approaches successfully enabled capture, recovery and characterization of immunoglobulins and influenza antigen(s) in complex with the immunoglobulins. However, PEG precipitation has the advantage of simplicity and is more suited for large scale studies.

## Introduction

Immune complexes (ICs) are formed when antibodies bind to antigens. Antigen(s) may be endogenous as occurs in autoimmune disorders or exogenous (i.e. drugs, toxins or infectious agents). ICs are formed during many inflammatory and infectious processes, and an extensive literature documents their pathophysiological role in disease processes, as we have previously reviewed [1]. ICs are cleared from the circulation by uptake into cells following binding of the immunoglobulin heavy chain Fc region to Fc gamma receptors (FcγRs) expressed by numerous cells (e.g. mast cells, monocytes, macrophages, neutrophils and basophils) [2]. The binding of ICs to FcγRs may lead to either activation or suppression of inflammatory cells depending on the type of FCGR gene expressed [2], and IC-induced cellular activation or suppression has been documented in many disease processes [3, 4]. ICs may also precipitate in tissues, leading to local inflammation or organ damage, influx of inflammatory cells and complement activation [5]. Their importance is well established in renal diseases [6]; inflammatory disorders such as rheumatoid arthritis [7] and systemic lupus erythematosus; infectious diseases such as dengue, sclerosing panencephalitis, hepatitis and malignancies [5, 8]. Although many studies report identification of ICs during disease processes, only a few have reported identification of antigens within ICs [9–12].

ICs may contain any class of immunoglobulin but mainly comprise IgM, IgA and IgG [5]. The major immunoglobulin class that is responsible for agglutination is IgM [5]. Methods for IC detection are typically based on their physicochemical properties, such as their size, antibody class, affinity for specific cellular receptors and susceptibility to phagocytosis by macrophages [13]. A variety of methods are available for isolation of ICs or identifying elements associated with but not directly identifying ICs and are summarized in Menikou *et al*. [1]. Examples include methods that are based on the attachment of ICs to specific cellular receptors such as Fc receptors, binding to platelets or macrophages, or binding to complement component 3 receptors on Raji cells. Other methods include binding of soluble complexes to C1q [5], or addition of radiolabelled anti-human IgG to determine the amount of bound ICs [14]. Further methods include detection of ICs by their ability to agglutinate platelets [15, 16], gel filtration using Sepharose 6B [17], or radiolabelled C1q in combination with polyethylene glycol (PEG) precipitation for removal of free C1q [18]. Methods using labelled aggregated IgG and labelled macrophages to study IC inhibition have also been used [19]. Examples of methods directly identifying ICs include purification by magnetic beads (Protein G or Protein A) with immobilized Protein G followed by nano liquid chromatography tandem mass spectrometry (LC MS/MS) analysis [9, 20–22]. However, when using magnetic beads there is the possibility of nonspecific binding of proteins and the sensitivity of detection of the antigens identified is considered low [9, 20].

ICs can be precipitated from serum using PEG [13], and it was later confirmed that low concentrations of PEG selectively precipitate ICs with minimal free IgG, probably by steric hindrance effects [23, 24]. Large proteins or complexes are precipitated by PEG by steric exclusion only if they are surrounded by linear PEG chains [25, 26]. Although numerous methods are available for recovery of ICs by PEG precipitation [5, 7, 23–32], very few studies have identified the antigen within ICs.

**Fig 1:**
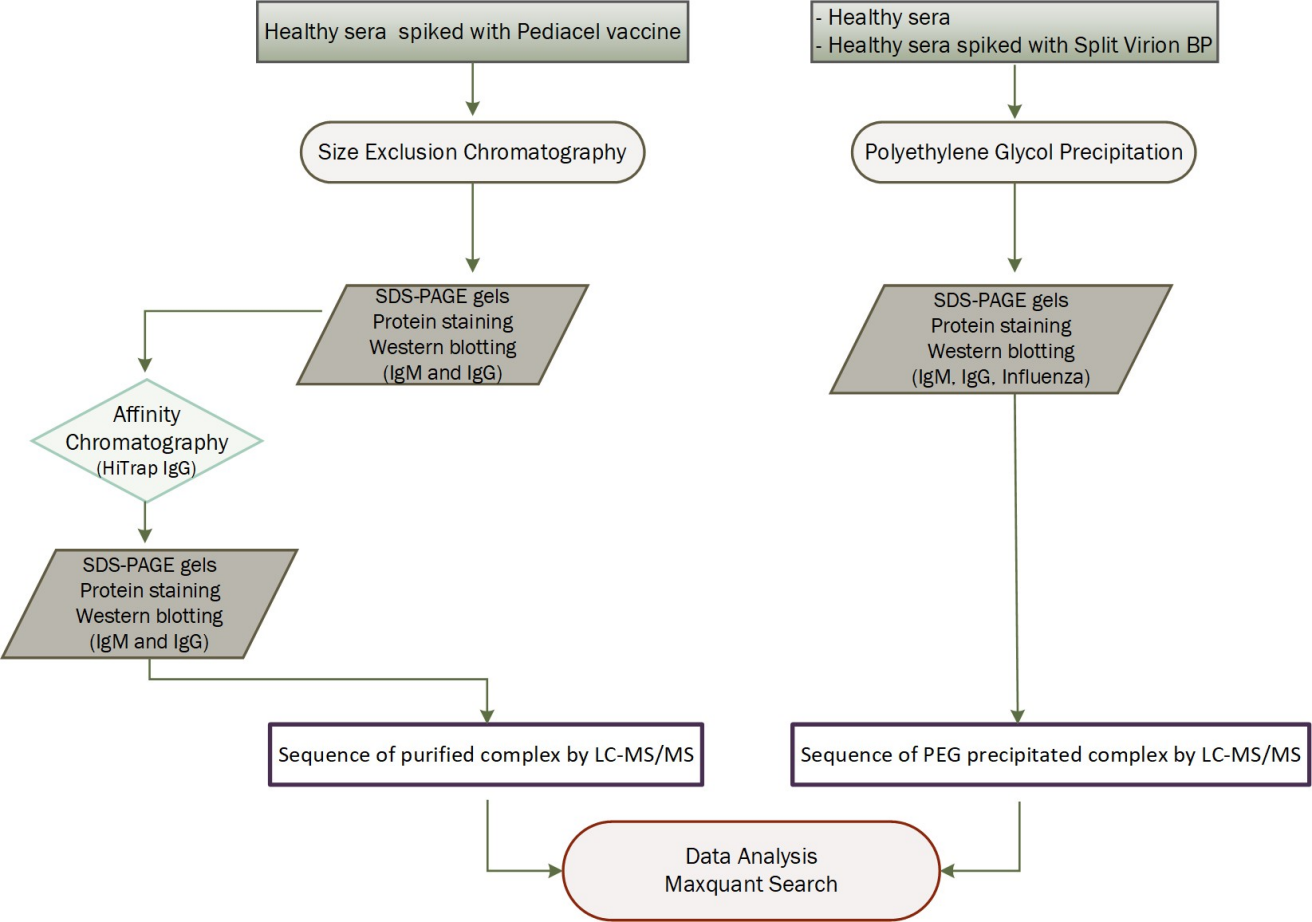
Experimental workflow showing the strategy involved to recover and purify ICs.

Here we present a method that enables recovery of ICs from serum, as well as the isolation and identification of the antigen that is bound to the ICs. We tested the performance of the method with artificially created complexes of immunoglobulin and influenza peptides, and compared size exclusion and affinity chromatography, versus conventional PEG precipitation for recovery of the ICs, linked to LC MS/MS for identification of the antigen (Fig 1). In both cases, we detected influenza proteins in the recovered fractions. We also characterised the immunoglobulin molecules and other proteins which were isolated.

## Materials and methods

### Sample collection

Serum was recovered from venous blood from seven healthy adult donors. Specific approval for the present study was given by Central Office for Research Ethics Committees, St Mary’s NHS Trust, U.K. under reference number REC 12/WA/0196 with an Imperial College Healthcare Tissue Bank project R13062. Verbal informed consent was obtained from healthy volunteers at the time of the experiments. All volunteers have subsequently given written acknowledgments of their verbal consent, and these acknowledgments have been filed along with a Note to File appended to the Investigator Site File and signed by the Investigator, which conforms to the ethics approval. All seven volunteers had been vaccinated with influenza vaccine Split Virion BP 2014/2015 (strains: A/California/7/2009(H1N1), A/Texas/50/2012(H3N2) and B/Massachusetts/2/2012 from Sanofi Pasteur) at 6–10 weeks prior to sample collection. In addition to the seven volunteers above, serum from two healthy individuals who had been immunized with the Split Virion BP 2015/2016 vaccine (A/California/7/2009 (H1N1)pdm09, A/Switzerland/971593/2013(H3N2), B/Phuket/3073/2013) 6 months prior to serum collection was used. Inclusion criteria for the volunteers were prior vaccination with influenza vaccine 6–10 weeks prior to sample collection, and exclusion criteria were that volunteers had no ongoing flu like or other illness, and were not taking any medication. The age range of the volunteers was 20–49 years of age. Ten patients with febrile illness within 10 days of onset of fever were recruited at University of California San Diego (UCSD) with parental consent and approval of the local research ethics committee (Human Research Protection Program #140220). Table 1 provides additional information about the demographic details of the febrile patients.

**Table 1:** Demographic details of the study patients.

Variable	Number of patients n = 10
**Gender**	
**Male, n (%)**	9 (90)
**Female, n (%)**	1 (10)
**Median Age**	2.8 (range 1.5–9.1)
**Ethnic background**	
**Caucasian, n (%)**	4 (40)
**Hispanic, n (%)**	2 (20)
**Asian, n (%)**	3 (30)
**Mixed, n (%)**	1 (10)

### Sample preparation

Volunteers’ venous whole blood was collected in serum-separating tube (SST) II advance bottles (Becton Dickinson), kept at room temperature (RT) for 30 minutes and centrifuged at 4°C at 3000 *g* for 10 minutes for serum separation and recovery.

In vitro ICs were created by adding 40 μl of influenza vaccine to 160 μl of serum. For PEG precipitation studies, two different volumes of influenza vaccine were evaluated—4 μl and 40 μl. Serum and influenza vaccine were mixed, incubated at 37°C for 1 hour to allow antibody-antigen binding, and then processed either by size exclusion chromatography (SEC) or PEG precipitation.

### Isolation of ICs from human sera

#### SEC

Serum spiked with influenza vaccine as above was fractionated on a Superdex 200 10/300 GL (GE Healthcare) pre-packed Tricorn column attached to an AKTA purifier 10 chromatography system (GE Healthcare). Samples were loaded and eluted in 50 mM NaPO_4,_ 150 mM NaCl (pH 7.0) buffer. Sample elution was monitored by 280 nm Ultraviolet (UV) absorbance. Molecular mass standards (High Molecular Weight Kit, GE Healthcare) were used to construct calibration curves. All fractions after the void volume (24 ml) were collected.

#### PEG precipitation

Equal 200 μl volumes of serum and 6% PEG 6000 (BDH) were mixed and dissolved in borate buffer comprising 100 mM boric acid (Sigma Aldrich), 75 mM NaCl (BDH), 25 mM sodium tetraborate (Sigma Aldrich), and incubated statically at 4°C overnight. The resulting precipitate was centrifuged at 2000 *g* for 20 minutes at 4°C. After decanting the supernatant, precipitates were washed once with a 400 μl PEG solution of the same final concentration (3%). Supernatant was decanted and the pellet dissolved in 200 μl of 1x phosphate buffer solution (PBS) (Sigma Aldrich) which was left to stand at 4°C overnight and stored at -80°C until analysed.

#### SDS-PAGE

Samples from PEG precipitation were separated on polyacrylamide gels; 3–8% Tris-acetate mini gels 1.5 mm (Life Technologies). Ten microliters of HiMark pre-stained high molecular weight (HMW) protein standard (Life Technologies) was used per gel to determine the molecular weight of the proteins that were loaded on the Tris-acetate mini gels.

#### Protein staining

After migration, gels were stained with Zinc reversible stain kit (Thermo Fisher Scientific) and then transferred to a nitrocellulose membrane for Western blotting.

#### Affinity chromatography

To further purify the proteins in the fractions collected by SEC, the two initial peaks of SEC were pooled individually and applied separately to a 5 ml HiTrap Protein G column (GE Healthcare) in 20 mM sodium phosphate buffer (pH 7.0). Samples were eluted with 0.1 M glycine-HCl (pH 2.7) buffer and neutralized with 1 M Tris-HCl buffer (pH 9.0).

#### Protein concentration

Centrifugal filter membranes (GE Healthcare) were prepared according to the manufacturer’s instructions. Membranes were rinsed in deionized water (Milli-Q water, HPLC grade, Acros). Vivaspin 20 membranes with 3,000 molecular weight cut-off (MWCO) were used. Each sample was centrifuged at 5,000 *g* for 15 minutes at 4°C using a swing bucket rotor.

#### Protein quantification

For total protein quantification of the resulting PEG precipitated samples, Coomassie Plus (Pierce) was used. The standard microplate protocol (working range = 100–1,500 μg/ml) was followed. Absorbance at 595 nm of each well was determined using a VERSAmax tunable microplate reader.

#### Western blotting

For detection of influenza proteins, samples were denatured and electrophoresed on 3–8% Tris-acetate gels (Thermo Fisher Scientific). Proteins were transferred onto polyvinylidene difluoride membrane (Thermo Fisher Scientific), probed with primary and secondary horseradish peroxidase (HRP)-conjugated antibody and then detected using enzyme chemiluminescent reagents (GE Healthcare). The primary antibody used for immunoblotting was rabbit polyclonal anti-H1N1 influenza A virus nucleocapsid protein (Ab104870, Abcam). The secondary antibody was goat anti-rabbit immunoglobulin HRP (P0448, Dako). Influenza vaccine (Split Virion BP 2015/2016) was used as a positive control.

#### Protein sequencing

Following protein quantification, the samples from affinity chromatography and PEG precipitation were sequenced by LC-MS/MS at the University of Bristol Proteomics Facility (UK) by in-gel digestion [33].

### Bioinformatic analysis

Mass spectrometry data were analysed using MaxQuant 1.6.6.10 [34]. In brief, spectra were searched against the UniProt human proteome (February 2019), immunoglobulin sequences (January 2018 Kabat and NCBI databases) from the abYisis database (Prof Andrew Martin, Personal Communication and [35]), and MaxQuant’s standard contaminant database. Trypsin was selected as the enzyme with full specificity and up to two missed cleavages. Peptide precursor mass tolerance was set at 10 ppm, and MS/MS tolerance was set at 0.5 Da. Search criteria included carbamidomethylation of cysteine (+57.0214) as a fixed modification and oxidation of methionine (+15.9949) as a variable modification. Intensity based absolute quantification (iBAQ) was employed. Match-between-runs was activated with default settings. False discovery rate was set at 0.01 for peptides and 1 for proteins (to prevent exclusion of immunoglobulin variable peptides which would be distributed between many protein sequences).

Downstream analysis was undertaken in R for Windows 3.5.1 [36]. Full gene names for UniProt proteins were determined by InterMineR. Individual proteins were classified using string matching as keratin, trypsin, complement, albumin, fibrinogen, immunoglobulin, other, contaminant and influenza. Individual peptides were then classified according to their corresponding protein(s). Where multiple classes matched, the first in the list preceding was selected.

iBAQ normalises total MS1 intensity per protein based on the number of tryptic peptides, approximating molar ratios of proteins within samples. We sought to calculate mass ratios and recalculate immunoglobulin results using constant region peptides only. We therefore calculated modified iBAQ-based values at peptide level.

Immunoglobulin peptides were classed as belonging to the constant region based on edit distance of two or below to a UniProt human constant immunoglobulin sequence (approximate Levenshtein distance algorithm).

For each protein, including immunoglobulin constant sequences, the number of tryptic peptides per kDa was calculated. Normalised peptide intensities were calculated by dividing the original intensities by the corresponding ratios. Non-constant region immunoglobulin peptide intensities were set to zero. Normalised constant immunoglobulin peptide intensities were then multiplied by the ratio between the molecular weight (MW) of the (sub)class and its constant region. Where a peptide corresponded to multiple immunoglobulin subclasses, the mean of each correction was taken.

Normalised intensities were summarised by class and sample, with the total for each sample normalised to correspond to the proportion of MS1 intensity belonging to identified features. In this way, differences in identification rates between samples remains informative.

The mass spectrometry proteomics data have been deposited to the ProteomeXchange Consortium via the PRIDE [37] partner repository with the dataset identifier PXD021575 and 10.6019/PXD021575.

## Results

### Analysis of protein complex formation by SEC, SDS-PAGE gels, protein staining and Western blots

**Fig 2:**
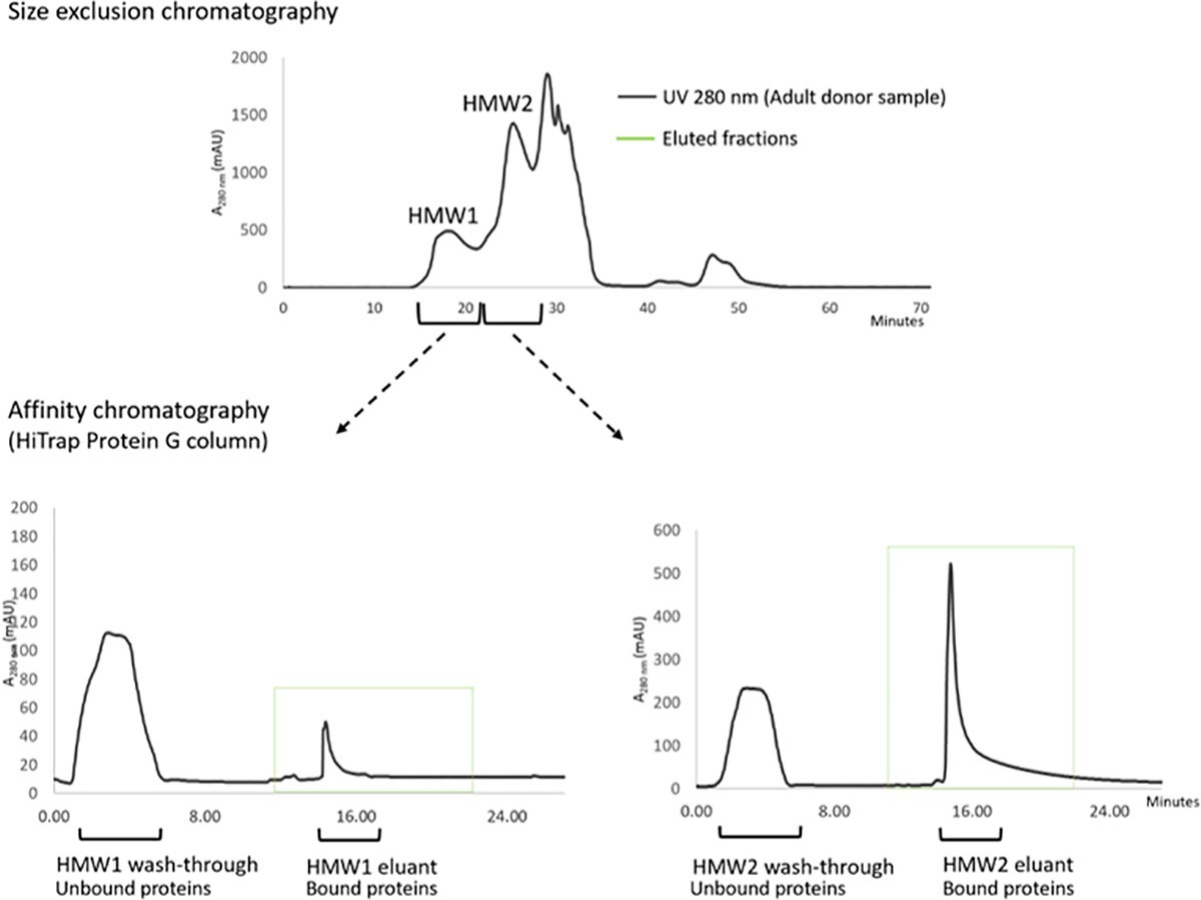
Experimental workflow used to recover and purify each peak from the affinity purification step prior to protein sequencing.

SEC of the mixed serum with influenza revealed a consistent pattern of peaks representing the eluted proteins on the chromatogram; a representative one is shown in Fig 2. Three major peaks were identified: a peak at c. 600 kDa (HMW1), a separate peak >150 kDa (HMW2), and an albumin peak (67 kDa). SDS-PAGE followed by Western blotting for IgG and IgM detection showed that the HMW1 peak mainly contained IgM, and the HMW2 contained IgG. However, IgG was also detected within HMW1, suggesting the presence of ICs in HMW complexes.

### Affinity chromatography of selected fractions from SEC

To further purify the IgM, IgG and other possible antigens bound to the immunoglobulins prior to peptide sequencing, affinity chromatography was undertaken. A pool of fractions from SEC was generated that corresponded to the HMW1 and HMW2 peaks and each pool was applied to a HiTrap Protein G column (Fig 2). Unbound proteins were washed off, and bound proteins recovered by pH 2.7 elution buffer (0.1 M glycine-HCl). Both the bound and eluted fractions were collected, the eluted fractions were neutralized to restore pH to 7.3 and then concentrated to original volume.

### Mass spectrometry analysis and data evaluation

Four pools of fractions of the recovered proteins were made from the affinity chromatography fractionation: bound and unbound fractions of the HMW1; bound and unbound pools of the HMW2 peaks (Fig 2).

After dialysis and concentration of the pooled fractions to their original volume, in-solution digestion was carried out on 1 μg of protein and the samples analysed by LC MS/MS.

### Identification of influenza antigens by Western blotting after SDS-PAGE

After denaturation and separation of the PEG precipitated proteins in 3–8% Tris-acetate mini SDS-PAGE gels, proteins were visualized by Coomassie Blue staining and compared with the protein profile of unprecipitated serum, and the influenza vaccine proteins.

**Fig 3:**
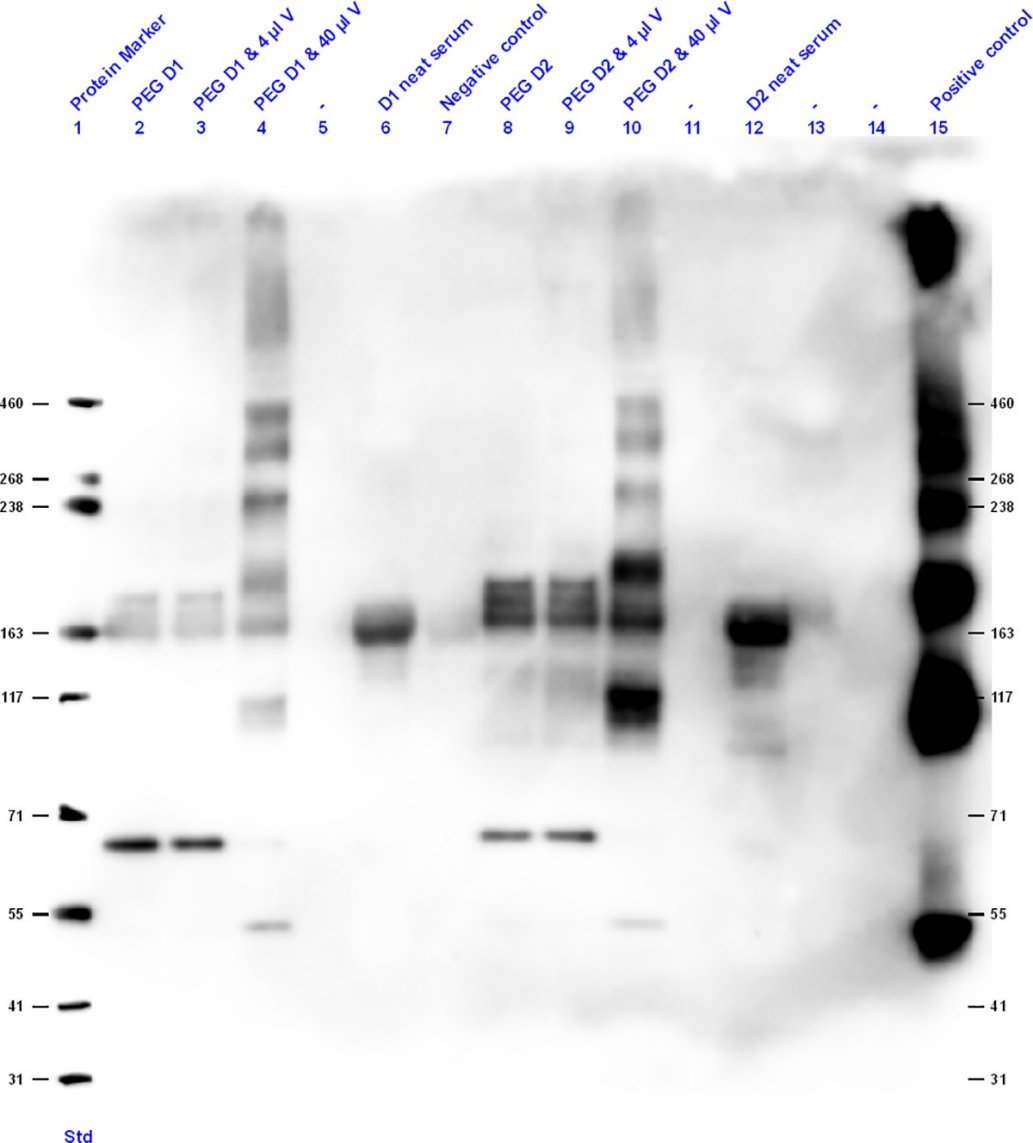
H1N1 Western blotting of PEG precipitates from two healthy adult control samples, healthy adult sera, PBS as a negative control and flu vaccine as a positive control, showed that PEG can precipitate in vitro ICs. Lane one is the Precision Plus protein standard ranging from 31 to 460 kDa. Three μg of protein per lane was loaded on a 3–8% Tris-acetate denaturing SDS-PAGE gel, blotted to nitrocellulose membrane and incubated with rabbit anti-H1N1 influenza A virus (primary antibody), goat anti-rabbit Immunoglobulin HRP (secondary antibody) and stained using the ECL kit. D stands for Donor, V for Vaccine and Negative control (Lane 7) is PEG with PBS. Lane 15 is a positive control, the influenza vaccine.

Fig 3 shows that influenza proteins were only detected in influenza vaccine spiked PEG precipitated samples, and in PEG precipitated samples where the concentration of the vaccine was highest (40 μl, Lanes 4 and 10). Additional bands that were present in the control samples are due to non-specific reactivity because the primary antibody is raised in rabbit and has IgG as an isotype.

**Fig 4:**
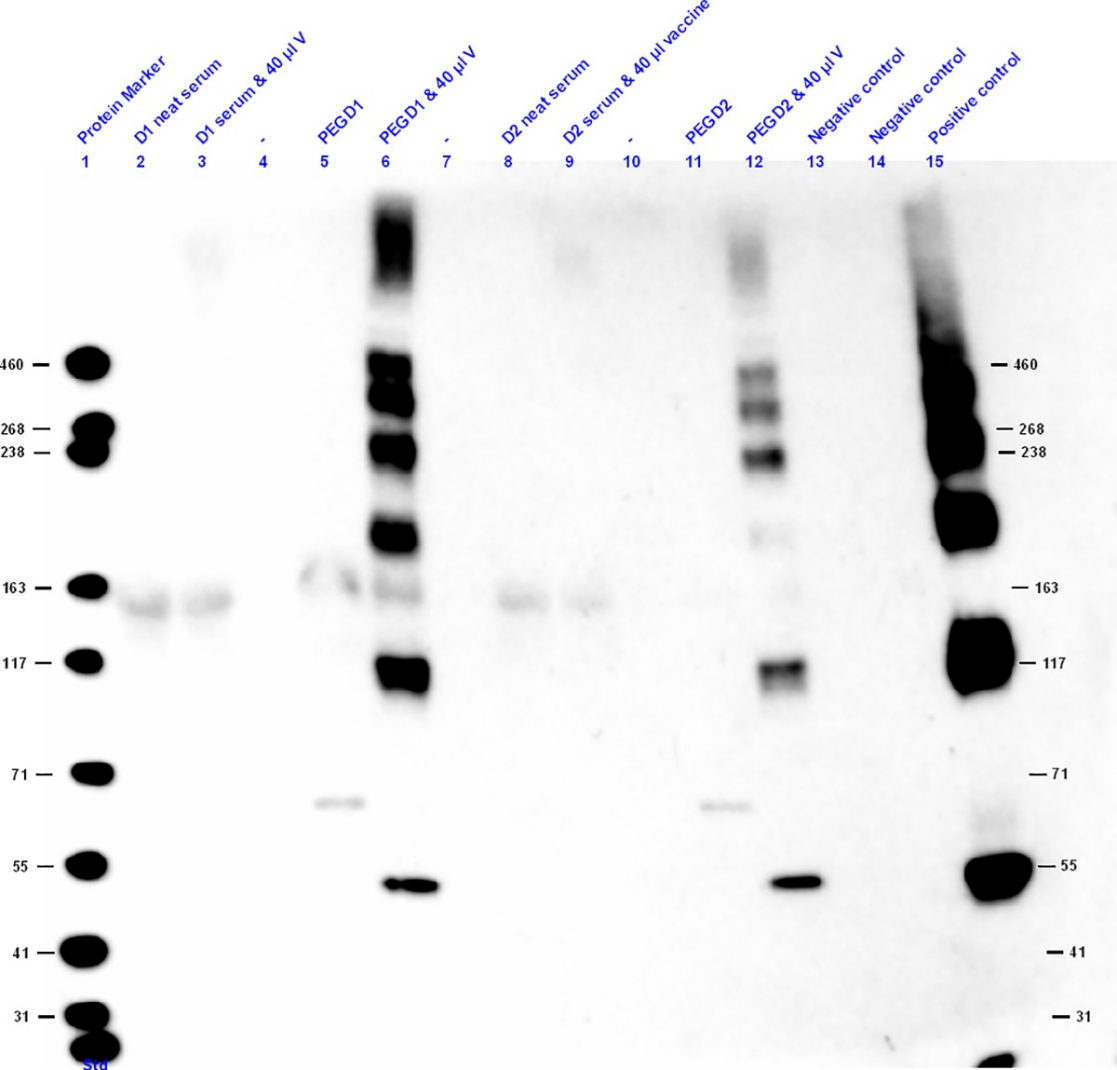
H1N1 Western blotting of healthy adult serum and serum spiked with vaccine, PEG precipitates from two healthy adult donor samples showed that influenza antigen was only detected in the PEG precipitated serum samples spiked with vaccine. Lane 1 of the gel is the Precision Plus protein standard ranging from 31 to 460 kDa. Three μg of protein per lane was loaded on a 3–8% Tris-acetate denaturing SDS-PAGE gel, blotted to nitrocellulose membrane and incubated with rabbit anti-H1N1 influenza A virus (primary antibody), goat anti-rabbit immunoglobulin HRP (secondary antibody) and stained using the ECL kit. D stands for Donor, V for vaccine. Negative control (Lane 13) is PEG with H_2_0 and 40 ul of vaccine and negative control (Lane 14) is PEG with PBS.

Having shown that influenza antigens can be detected by Western blotting in PEG precipitated vaccine spiked in two donor serum samples (Fig 3, Lanes 4 and 10), additional experiments were performed to compare the detection of influenza antigens in vaccine spiked serum without PEG precipitation. As shown in Fig 4, influenza antigen was detected by Western blotting only in the serum samples spiked with influenza vaccine that were subjected to PEG precipitation (Lanes 6 and 12), and not when the unprecipitated serum spiked with same concentration of vaccine was used, nor in the unspiked (Lanes 5 and 11) or negative controls (Lanes 12 and 13).

These Western blotting experiments using anti-H1N1 antibodies (Fig 3), confirmed that PEG precipitation can capture influenza proteins in ICs (Lanes 4 and 10), and none of the negative controls (unspiked serum) showed detectable bands (Lanes 2 and 8). Serum that was spiked with 40 μl of vaccine and not subjected to PEG precipitation had barely detectable bands. Thus, we concluded that PEG precipitation concentrated the influenza proteins during the precipitation of the ICs.

### Mass spectrometry analysis

#### 1. Identification of protein classes

**Fig 5:**
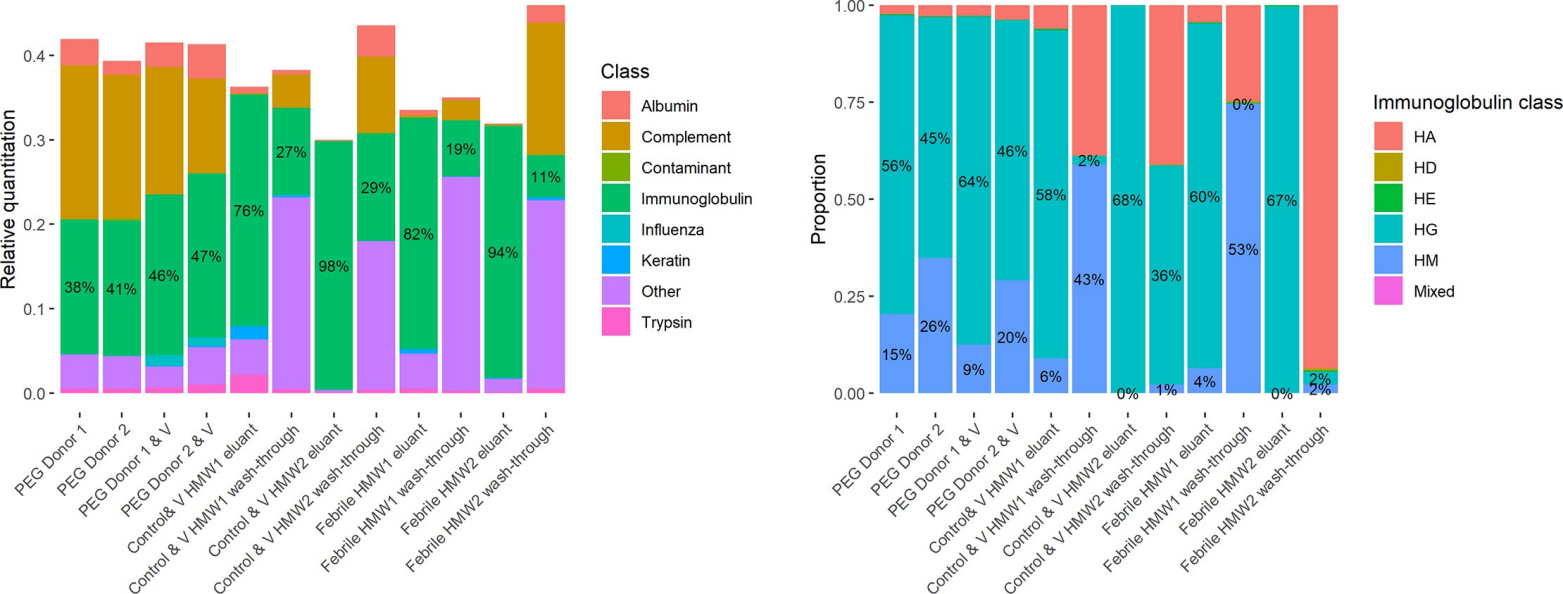
Quantification of different protein classes and identification of immunoglobulins. V indicates samples to which influenza vaccine was added. HMW1 refers to the highest molecular weight peak and HMW2 refers to the second peak. Febrile HMW1 eluant, wash-through and Febrile HMW2 eluant and wash-through are not spiked with vaccine. (A) Relative quantities of different classes of protein as calculated through a modified iBAQ-based approach. The total height of each bar is the proportion of MS1 intensity belonging to each identified feature. Samples come from two donors (Donor 1 and Donor 2). (B) Proportion of immunoglobulin mass by class as estimated using a modified iBAQ-based approach and extrapolated from constant peptides only. HA: IgA heavy chain, HD: IgD heavy chain, HE: IgE heavy chain, HG: IgG heavy chain and HM: IgM heavy chain.

We applied a modified iBAQ approach to obtain relative mass-based quantification of different classes of protein in the samples (Fig 5A). The proportion of MS1 intensity belonging to identified features is lowest (around 30%) in the samples with the highest proportion of immunoglobulin (Control & V HMW2 eluant and Febrile HMW2 eluant columns in Fig 5A), which is expected due to the limited feature identification expected for variable immunoglobulin peptides.

The highest proportion of immunoglobulin is identified in the HMW2 peak from SEC that was eluted from the affinity column (94% and 98%). The unbound fractions have low proportions of IgG (11% and 29%) indicating that the HiTrap Protein G column efficiently bound IgG. Complement effectively passed into the wash-through (0.2% and 0.5% in eluant vs. 21% and 34% in wash-through).

The HMW1 eluants, which should be enriched for IgM and ICs also had high proportions of immunoglobulin (76% and 82%), and the wash-throughs were similarly depleted (19% and 27%). Complement components were in low abundance overall and mostly passed into the eluant (0.4% and 0.9% vs. 7% and 10%).

The PEG precipitates contained intermediate proportions of immunoglobulin (38–47%) with complement being the other major constituent (27–44%). The proportion of immunoglobulin was increased in the vaccine-spiked samples compared to controls (46% and 47% vs. 38% and 41%).

IgG dominated in the HMW2 eluant (99.4% and 99.7%) and was nearly absent in the febrile wash-through (2%) (Fig 5B). In the influenza spiked wash-through a larger proportion of immunoglobulins was IgG. This suggests that the column may have been saturated. The remainder in both cases was predominantly IgM. IgG also dominated in the HMW1 eluants (85% and 89%), with IgM (6% and 9%), and IgA (4% and 6%) present. The wash-throughs were dominated by IgM and IgA.

**Fig 6:**
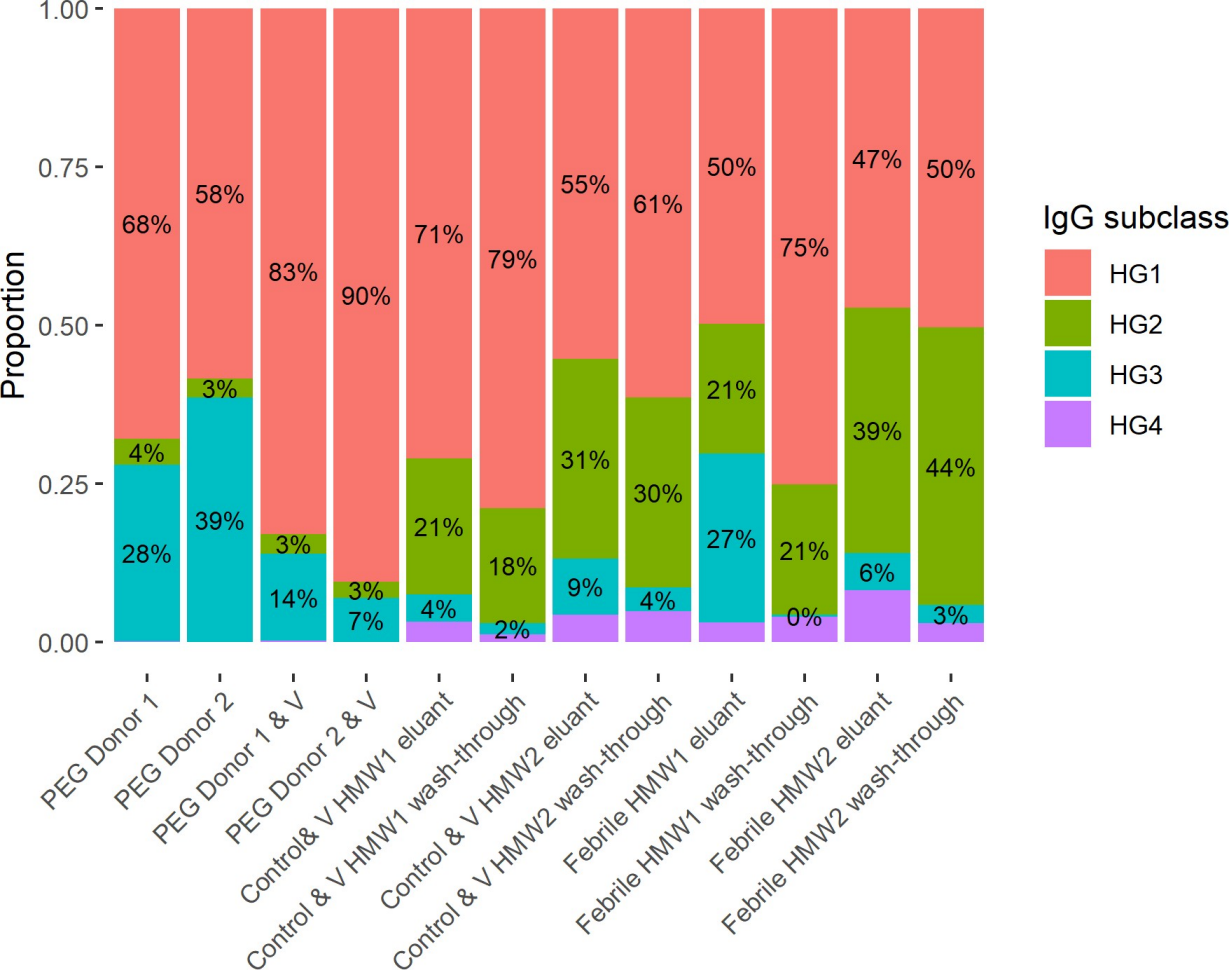
Proportion of estimated IgG mass by subclass (excluding contributions by non-specific peptides). PEG precipitated samples come from two donors (D1 and D2) and V indicates samples to which influenza vaccine was added. HMW1 refers to the highest molecular weight peak and HMW2 refers to the second peak. Febrile HMW1 eluant, wash-through and Febrile HMW2 eluant and wash-through are not spiked with vaccine. HG1: heavy chain of IgG subclass 1, HG2: heavy chain of IgG subclass 2, HG3: heavy chain of IgG subclass 3 and HG4: heavy chain of IgG subclass 4.

The identification of IgG subclasses (HG1, HG2, HG3, HG4) was further explored (Fig 6). Some peptides could not be uniquely assigned to a subclass (accounting for 51–80% of estimated mass). IgG1, as expected, was in the greatest abundance across all samples. Notably, in the PEG precipitates the proportion of IgG1 was increased in the influenza vaccine spiked samples vs the PEG Donor 1 and 2 samples that were not spiked (83% and 90% vs. 68% and 58% respectively).

#### 2. Identification of Influenza proteins

**Fig 7:**
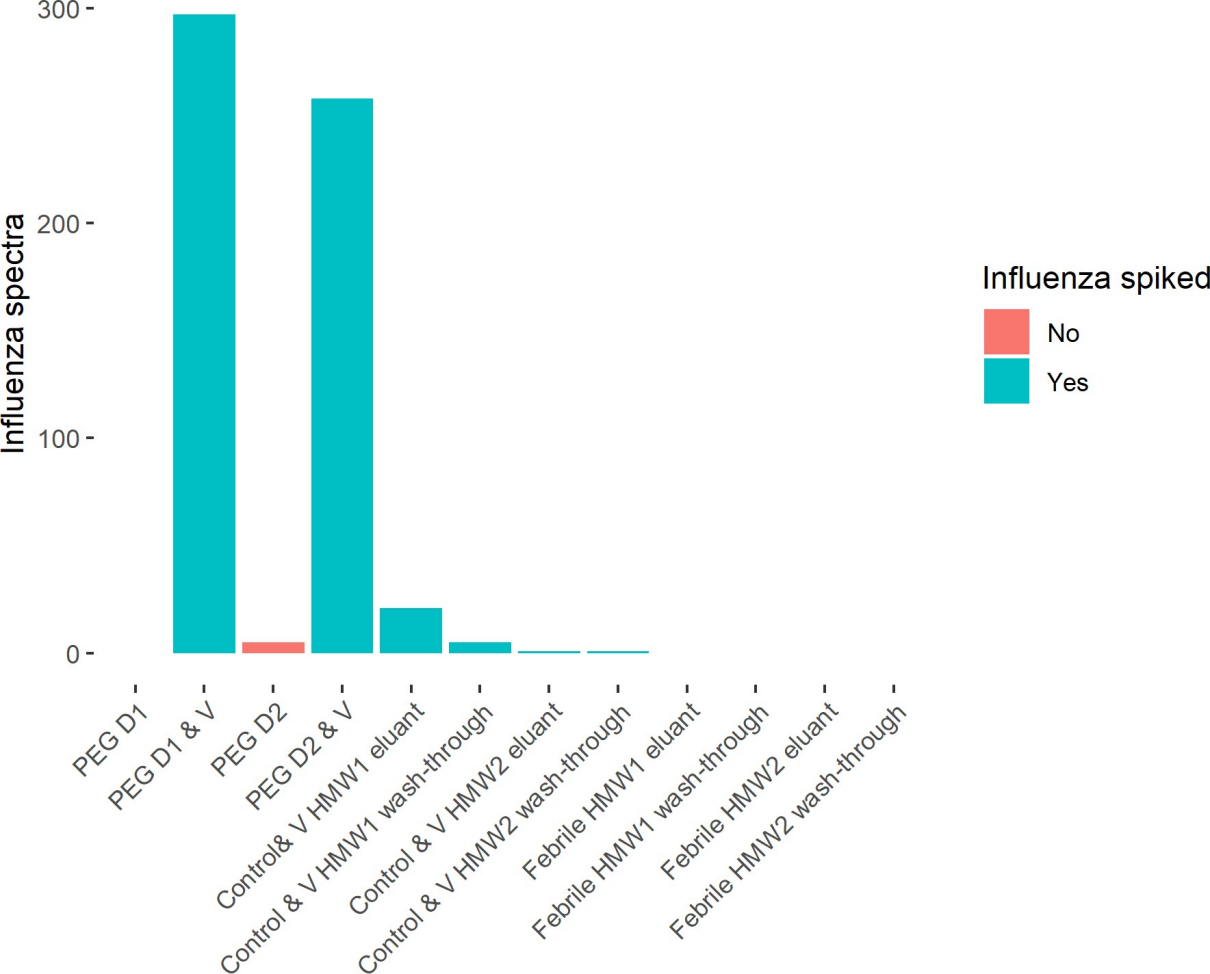
**Number of MS2 spectra assigned to influenza peptides in each sample.** MS1 features matched by run are excluded. Samples come from two donors (D1 and D2). V indicates samples to which influenza vaccine was added. SEC-AP V HMW1 eluant, wash-through, and SEC-AP HMW2 eluant and wash-through are the control samples spiked with vaccine. SEC-AP Febrile HMW1 eluant, wash-through and SEC-AP Febrile HMW2 eluant and wash-through are not spiked with vaccine.

Influenza peptides were detected in all vaccine-spiked samples (Fig 7), the greatest number of spectra being in the PEG precipitates. More spectra mapped to influenza peptides were identified in the eluant of the HMW1 eluant fraction, suggesting they had been captured in ICs. The estimated mass contribution of influenza protein was also higher in the spiked PEG samples (2.4% and 3.2%) than the HMW1 eluant (0.02%). The five spectra that matched to influenza in unspiked samples corresponded to high abundance influenza peptides from spiked samples, suggesting some potential sample crossover.

## Discussion

The pipeline of SEC followed by affinity chromatography and LC MS/MS successfully recovered and identified spiked influenza peptides. Influenza peptides were not identified in the non-spiked febrile controls apart from five peptides in one fraction, most likely carry over from the spiked sample. The healthy adult controls spiked with influenza vaccine were used to confirm that it is possible to recreate *in vitro* ICs and identify the antigen within the IC.

Protein sequencing by LC MS/MS detected influenza peptides indicating that the procedure followed for recreation of ICs *in vitro* was successful. The results from experiments using sera from healthy adult controls previously immunised with influenza vaccine that were spiked with influenza vaccine to recreate ICs *in vitro*, identified the presence of influenza antigen in HMW pools from affinity chromatography. Identification of the influenza antigen principally in the highest molecular weight protein G purified fraction (HMW1) supports their presence and recovery from IC.

Although the combination of SEC and affinity chromatography resulted in highly enriched immunoglobulin fractions, with co-recovery of the influenza proteins bound to immunoglobulin, the procedure is slow and not suitable for high throughput analysis. We found the simpler PEG precipitation method, although precipitating other high molecular weight proteins, also successful recovered influenza proteins bound to immunoglobulin. Western blotting demonstrated that precipitation of influenza vaccine alone was limited. The PEG procedure is thus more suitable for analysis of large numbers of individual samples, whereas the SEC/affinity purification may be more suited for identifying antigens within a large pool of samples.

We have shown that our procedure can identify the influenza antigen within ICs produced *in vitro* after spiking the sample with the vaccine. This sequence of experiments can enrich and detect antigens within ICs.

There is controversy as to whether PEG preferentially precipitates only circulating ICs. Our results, showed that this is not the case and are consistent with other earlier studies [31]. In our results, there were PEG precipitated proteins and, in particular, IgM and IgG in samples, without *in vitro* ICs. In addition to immune proteins, a number of studies demonstrated that PEG precipitated sera not only contain free immunoglobulins and C3 but other proteins such as albumin and fibronectin [7, 38].

The general mechanism of the action of PEG is not yet understood but it can be used to precipitate proteins from complex mixtures. The experiments described in this paper show that this simple and well-established approach can be used to recover ICs and identify the antigen within the complexes. PEG precipitation is a simple method; it does not involve many steps that could introduce contamination and has potential advantages in searching for an unknown antigen. For example, PEG does not denature proteins as much as other organic solvents and the PEG is used in a low concentration (3%) as most of it is in the supernatant of the solution [26]. LC MS/MS results showed that PEG precipitation preferentially precipitates immunoglobulin and complement regardless of the presence of ICs. The addition of influenza vaccine resulted in greater precipitation of immunoglobulin and increased proportion of IgG1, consistent with formation of ICs, since it is known that the response to IIV (inactivated influenza vaccine) is mostly IgG1 [39–41].

In conclusion, we have shown that this procedure can identify the influenza antigen within ICs produced *in vitro* after spiking the sample with the vaccine. The procedure may be useful in identifying the target antigen in many diseases involving ICs.

## Supporting information

S1 Raw images (TIF)
