# Supplementary figures and images for: A proteomics-based method for identifying antigens within immune complexes
Source: PLoS One. 2020 Dec 23;15(12):e0244157. doi: 10.1371/journal.pone.0244157 (PMC7757895; doi:10.1371/journal.pone.0244157)

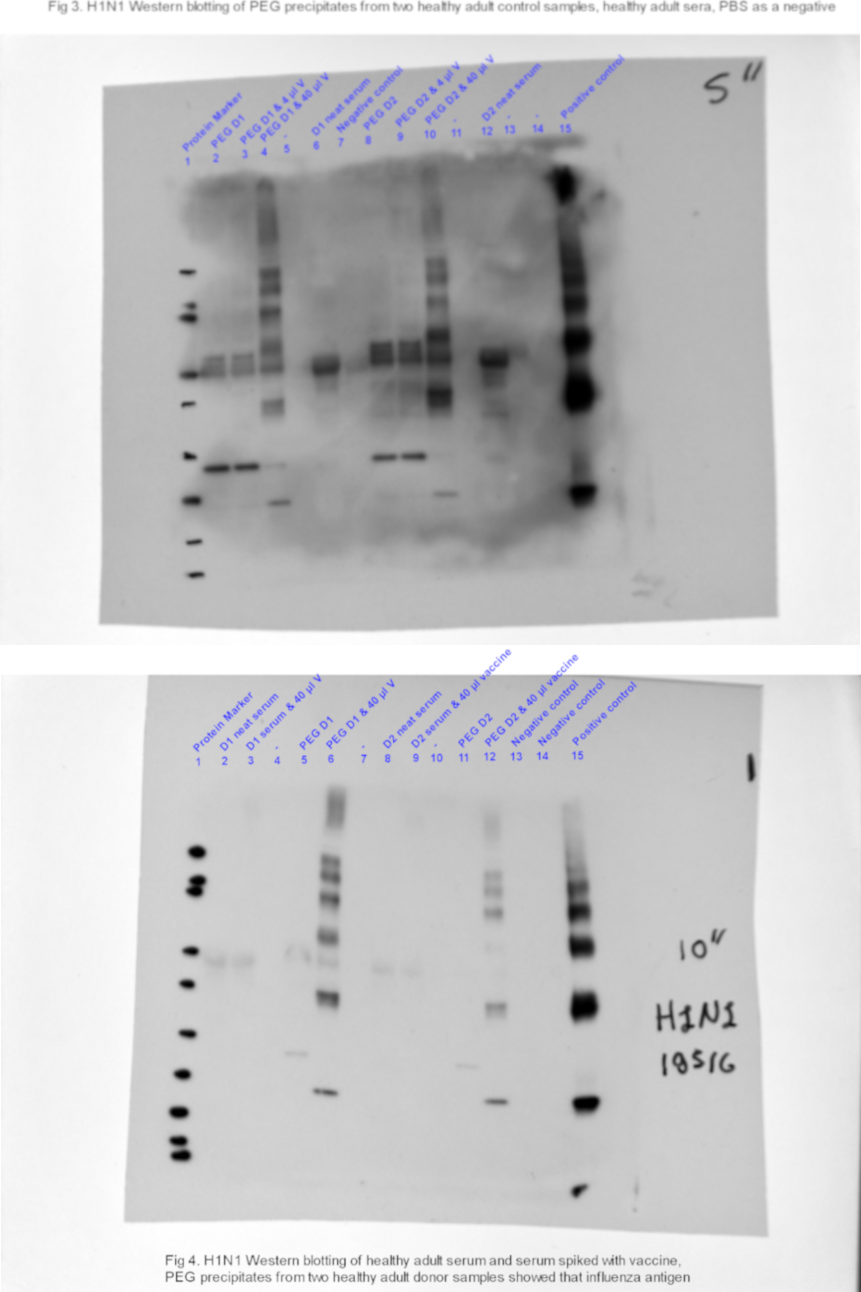

Supplement: S1 Raw images — (TIF) [file pone.0244157.s001.tif]
